# What Does Next-Generation Mass Spectrometry Offer for Proteomics? A Comprehensive Platform Comparison

**DOI:** 10.1021/acs.jproteome.5c01007

**Published:** 2026-03-26

**Authors:** Filipa Blasco Tavares Pereira Lopes, Daniela Schlatzer, Tara Sudhadevi, Anantha Harijith, Marzieh Ayati, Mehmet Koyutürk, Mark R. Chance

**Affiliations:** † Center for Proteomics and Bioinformatics, 2546 Case Western Reserve University, Cleveland, Ohio 44106, United States; ‡ Department of Nutrition, 2546 Case Western Reserve University, Cleveland, Ohio 44106, United States; § Department of Pediatrics, 2546 Case Western Reserve University, Cleveland, Ohio 44106, United States; ∥ Department of Computer Science, 12331 University of Texas Rio Grande Valley, Edinburg 78539, United States; ⊥ Department of Computer and Data Sciences, 2546 Case Western Reserve University, Cleveland, Ohio 44106, United States

**Keywords:** data-independent acquisition, DIA, data-dependent acquisition, DDA, Orbitrap Astral, timsTOF Ultra, bronchopulmonary dysplasia, proteomics

## Abstract

Next-generation mass spectrometry platforms (Orbitrap Astral, timsTOF Ultra) are reshaping proteomics by enhancing analytical depth and sensitivity. We compared these platforms against Orbitrap Exploris 480 using neonatal mouse lung tissues from a bronchopulmonary dysplasia model (*n* = 12), employing four acquisition strategies: Exploris 480 DDA/DIA, Astral HR-DIA, and timsTOF Ultra DIA-PASEF. All platforms identified ∼4000 proteins in common, with 98% proteome coverage of data-dependent acquisition (DDA) identifications using data-independent (DIA) methods and 92% concordance between next-generation systems. Orbitrap Astral and timsTOF Ultra quantified >225,000 peptides and 13,000 proteins, representing ∼800% and ∼300% greater depth than Exploris 480 DDA, respectively. Furthermore, new-generation platforms cut recommended sample size by ∼66%. Enhanced proteome depth improved subcellular compartment annotations from 30% (DDA) to 66% (next-generation platforms) and reactome pathway coverage from 58% (DDA) to 90% (next-generation platforms). Differential expression analysis identified up to four times more phenotype-associated proteins in DIA data sets, enriched in mitochondrial, ribosomal, and extracellular components, with up to 44 enriched pathways. Importantly, proteins uniquely detected showed no functional annotation bias. These findings demonstrate that DIA acquisition on multivendor next-generation platforms provides superior proteome coverage and more complete systems biology assessment without introducing bias, enabling enhanced understanding of complex biological systems.

## Graphical abstract

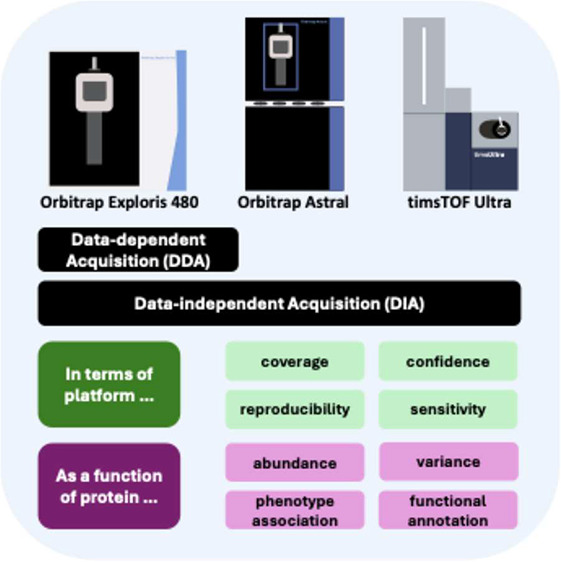

## 1 Introduction

Bottom-up proteomics is the premier method for providing high throughput and reliable characterization of the proteome.^1,2^ Data acquisition strategies include data-dependent acquisition (DDA) and data-independent acquisition (DIA). Since the early 2000s, DDA has been the gold-standard for bottom-up proteomics; a decade later DIA methods started being developed with help of mass spectrometry (MS) instruments and bioinformatic tools.^3–6^ These methods differ in the isolation and selection of precursor ions for MS2 fragmentation. In DDA, precursor ions are typically selected based on MS1 intensity and sequentially fragmented generating individual fragmentation spectra that are used for sequencing, while in DIA, the instrument scans a set of predefined mass ranges, and all precursors are simultaneously fragmented. The fragmentation data generated from DIA are very complex and have historically been challenging to analyze. Recent advances in data acquisition and informatic applications have enabled reliable quantification and robust sequencing of peptides, overcoming many challenges faced by DIA.^6–8^ These advances have improved throughput, quantification, and proteome coverage providing deep proteome profiling in label-free analyses.^9,10^

As DIA techniques advance and became practical, instruments have also advanced; next-generation mass spectrometers with improved sensitivity and scan rates can quantify 10,000s of proteins in a single analysis using DIA. Two such examples are the Thermo Orbitrap Astral and the Bruker timsTOF Ultra, both released in 2023. The Orbitrap Astral is a novel high resolution mass spectrometer that pairs a quadrupole Orbitrap mass spectrometer with the asymmetric track lossless analyzer (Astral). The Astral uses a high-speed extraction trap to accumulate ions and incorporates 3-dimensional ion focusing, asymmetric ion mirrors, and ion foils to enhance and shape the ion path, thereby precisely aligning ions in the analyzer. These unique features maximize transmission and sensitivity by achieving nearly lossless ion transfer.^11^ Coupled with ultrafast acquisition rates (up to 200 Hz), this instrument provides better scan times, resulting in ultrafast sequencing speeds and a large dynamic range. The timsTOF platform utilizes gas phase ion separation via trapped ion mobility spectrometry (TIMS) coupled to an ultrahigh resolution quadrupole time-of-flight mass spectrometer. This adds an additional dimension of separation based on size and shape (collisional cross section).^12,13^ In addition, the dual TIMS tunnel device sorts and time-focuses ions prior to the QTOF mass analyzer. Parallel accumulation-serial fragmentation PASEF capitalizes on the use of a dual TIMS device. Ions are collected simultaneously in the first TIMS tunnel and separated by size and shape in the second. As ions are emitted from the second tunnel, a second set of ions is collected in the first tunnel, preventing the loss of precursor ions and thereby significantly improving the duty cycle.^14^ The release of these ions from the TIMS device is synchronized with the precursor fragmentation in the QTOF mass analyzer. This dramatically increases sequencing speeds while retaining maximum resolution for both MS1 and MS2 scans.^15,16^ As with the Astral, the improved sequencing speeds in the timsTOF platform have transformed DIA for label-free protein expression, enabling improved detection and quantification of the proteome when compared to previous Orbitrap instrumentation.^17–19^

Although there have been studies on benchmarking these new instruments, most only compare one of them against a legacy platform. In an effort to provide a comprehensive evaluation, we have benchmarked DIA acquisition in both the Astral and timsTOF Ultra against the 480 Exploris in DDA and DIA modes. Previous studies have evaluated the performance of new-generation instruments with the goal of aiding method development (e.g., compatible with paraffin-embedded tissues, narrow-window DIA optimization, single-cell from low-input samples, low-cost 10$ proteome, 1 h proteome, and round-robin study to evaluate the profiling and reproducibility of clinical samples).^9,17,18,20–23^ Our study focuses on providing a systems biology evaluation, focusing on how increased proteome depth impacts biological interpretation. To harp on the biological interpretability of the data, we utilized an animal neonatal mouse lung tissue from an animal model of bronchopulmonary dysplasia in room air/normoxia (NO) and hyperoxia (HO) environments with the same samples analyzed across all instruments.

## 2 Experimental Section

### 2.1 Collection of Mouse Lung Tissues for Mass Spectrometry

All animal procedures using mice were approved by the Institutional Animal Care and Use Committee of Case Western Reserve University, Cleveland (Protocol No. 2020-0052). Newborn C57BL/6 mice (both male and female) were used in this study. Within 12 h of birth, pups were randomized to either normoxia (NO, 21% O2) or hyperoxia (HO, 85% O2) groups. Oxygen and air were mixed to maintain specific oxygen levels, which were verified using an oxygen monitor (MiniOX 200E, OhioMedical) placed inside the chamber. Hyperoxia exposure was maintained for 14 days in a sealed chamber with controlled oxygen levels, temperature, and humidity.^24,25^ Lactating dams were switched between the NO and HO cages every 24 h. Following exposure to NO or HO, the pups were euthanized on postnatal day 14. An equal number of male and female mice (3 mice/group) were used for all experiments. 5 mg of lung tissue samples from NO and HO mice were isolated, lysed, and cleaned-up using EasyPep MS Sample Prep Kits (Thermo Fisher, cat #A45733) following manufacturer’s instructions. Protein concentration was determined using the Bio-Rad Protein Assay Kit (BSA) (Bio-Rad, cat #5000002). Samples were normalized and separated into four aliquots, one for each MS platform analysis (Orbitrap Exploris 480—DDA, Orbitrap Exploris 480—DIA, Orbitrap Astral, and timsTOF).

### 2.2 Mass Spectrometry

#### 2.2.1 Orbitrap Exploris 480 with DDA

Samples were normalized to 600 ng of digest, blinded, and randomized for LC–MS/MS acquisition using Vanquish NanoUPLC (Thermo Fisher, USA) coupled with a Thermo Fisher Orbitrap Exploris 480 mass spectrometer via a Nanospray ion source (Thermo Fisher, USA). 600 ng of peptides was loaded onto a 15 cm × 75 μm PepMap column (Thermo Fisher, USA) and separated at a flow rate of 300 nL/min in direct injection mode using a Vanquish NanoUPLC (Thermo Fisher, USA). Mobile phase A was 0.1% formic acid (FA) in water, and mobile phase B was 0.1% FA in 98% acetonitrile in water. Samples from whole-proteome digests were acquired with a gradient starting with a linear increase from 1 B to 40% B over 168 min. The column was equilibrated using 4 volumes of solvent A (0.1% formic acid in water). The MS was operated in DDA mode by using a Thermo Fisher Orbitrap Exploris 480 mass spectrometer with the following parameters: MS1 mass resolution of 120,000, MS1 scan range of 375–1600 (*m*/*z*), charge state of 2–6, 1.6 (*m*/*z*) mass window for precursor ion isolation, HCD with a collision energy of 30%, dynamic exclusion (exclusion after 1 scan and a duration of 20s), absolute AGC value of 1e5, maximum injection time of 30 ms, and 1 microscan. Orbitrap Exploris 480 with the following DIA: Samples were normalized to 600 ng of digest, blinded, and randomized for LC–MS/MS acquisition using a Vanquish NanoUPLC (Thermo Fisher, USA) coupled with a Thermo Fisher Orbitrap Exploris 480 mass spectrometer via a Nanospray ion source (Thermo Fisher, USA). 600 ng of peptides was loaded onto a 50 cm × 180 μm μPAC column (Thermo Fisher, USA) and separated at a flow rate of 300 nL/min in direct injection mode using Vanquish NanoUPLC (Thermo Fisher, USA). Mobile phase A was 0.1% formic acid (FA) in water and mobile phase B was 0.1% FA in 98% acetonitrile in water. Samples from whole-proteome digests were acquired with a gradient starting with a linear increase from 1% B to 25% B over 45 min, followed by a further linear increase to 37% B in 15 min. The column was equilibrated using 4 volumes of solvent A (0.1% formic acid in water). The MS was operated in DIA mode using a Thermo Fisher Orbitrap Exploris 480 mass spectrometer with the following parameters: MS1 mass resolution of 60,000, MS1 scan range of 400–1000 (*m*/*z*), HCD with a collision energy of 30%, MS2 fixed scheme, MS2 scan range of 400–1000 *m*/*z* in 49 isolation windows with 12 Th isolation width, MS2 mass resolution of 15,000, MS2 window overlap 1 *m*/*z*, 800% normalized AGC target, and max injection time set to auto. Orbitrap Astral: Samples were normalized to 175 ng of digest, blinded, and randomized for LC–MS/MS acquisition using a Vanquish Neo UHPLC (Thermo Scientific, USA) system coupled with an Orbitrap Astral mass spectrometer (Thermo Scientific, USA) via an EASY-Spray Source (Thermo Scientific, USA). 175 ng of peptides was loaded onto an Aurora Frontier 60 cm × 75 μm C18 UHPLC column (IonOpticks, Australia) and separated at a flow rate of 350 nL/min in direct injection mode using a Vanquish Neo UHPLC system (Thermo Scientific, USA). Mobile phase A was 0.1% formic acid (FA) in water, and mobile phase B was 80% acetonitrile with 0.1% FA in water. The peptides were separated using a step gradient of 4–30% B over 45 min, followed by 30–45% B over 15 min. Eluted peptides were ionized at +2000 V using an EASY-Spray Source (Thermo Scientific, USA) and analyzed on an Orbitrap Astral mass spectrometer (Thermo Scientific, USA). The ion transfer tube temperature was set to 290 °C, and the RF was set to 40%. MS1 spectra were acquired on the Orbitrap mass analyzer with a resolution of 240,000 and a scan range of 380–980 *m*/*z*. The AGC target was set at 500%, and the maximum injection time was set at 5 ms. Precursor ions were fragmented through the HCD with a normalized collision energy (NCE) of 25%. Fragment ions were analyzed on the Astral mass analyzer with an isolation window of 2 Th from 380 to 980 *m*/*z*. For MS2 acquisition, the MS2 fixed scheme was applied with a scan range of 380–980 *m*/*z*, using 299 isolation windows of 2 *m*/*z* isolation width. The AGC target was set at 500%, and the maximum injection time was set at 3.5 ms. timsTOF Ultra: Samples were normalized to 175 ng of digest, blinded, and randomized for LC–MS/MS acquisition using an NanoElute 2 (Bruker Daltonik, Germany) system coupled online to a hybrid TIMS-quadrupole TOF mass spectrometer operating at full resolution at MS1 and MS2 levels (timsTOF Ultra^15^ Bruker Daltonik, Germany) via a Captive Spray Ultra nanoelectrospray ion source (Bruker Daltonik, Germany). Samples were normalized to 175 ng of digest, blinded, and randomized for LC–MS/MS acquisition using an Aurora column (25 cm × 75 μm ID, 1.7 μm reversed phase; Ion Opticks, Australia) at a flow rate of 250 nL/min in an oven compartment heated to 50 °C. Samples from whole-proteome digests were acquired with a gradient starting with a linear increase from 5% B to 25% B (0.1% formic acid in acetonitrile) over 48 min, followed by further linear increases to 37% B in 4 min and to 90% B in 4 min, which was held constant for 4 min. The column was equilibrated using 4 volumes of solvent A (0.1% formic acid in water). The timsTOF was operated in dia-PASEF mode^26^ with a mass range from 100 to 1700 *m*/*z*, 1/k0 start at 0.75 V s/cm2 and end at 1.30 V s/cm2, ramp and accumulation times set to 50 ms, capillary voltage of 1600 V, dry gas flow of 3 l/min, and dry temp of 200 °C. dia-PASEF settings were set to an optimized acquisition scheme using py_diAID software^27^ with dia-window isolation width starting from 7 to 95 Da. Each cycle consisted of 1x MS1 full scan and 60x MS2 windows covering 350–1250 *m*/*z* and 0.75–1.30 1/k0 (Table S1, Figure S1). The cycle time was 1.18 s. CID collision energy was 20 eV (0.60 1/k0) to 59 eV (1.6 1/k0) as a linear function of the inverse mobility of the precursor.

### 2.3 Mass Spectrometry Data Processing

#### 2.3.1 DDA Data Processing

Raw LC–MS/MS data were processed using Peaks v10.6 software (Bioinformatics Solutions, ON, CA) as described in.^28,29^ Peptide identification was performed using the UNIPROT database (UNIPROT_UP000000589, entries = 54,910, taxonomy = *Mus musculus* (mouse)). Protein identification was set to trypsin enzyme specificity and included carbamidomethylation as a fixed variation, methionine oxidation as a variable modification, and one missed cleavage. Label-free quantification was performed using default settings, which included a mass error tolerance of 10 ppm, a retention time shift tolerance of 5.0 min, 0.6 Da for fragment ions, TIC normalization, and an FDR threshold of 1%. Protein abundance was obtained by summing the three most intense unique peptides per protein. Imputation was performed using nonparametric missing value imputation using Random Forest^30^ (Table S2). DIA Data processing: Raw LC–MS/MS data were processed using directDIA mode on Spectronaut v19.3 ^31^ (Biognosys, Switzerland). Peptide identification was performed using the UNIPROT database (UNIPROT_UP000000589, entries = 54,910, taxonomy = *Mus musculus* (mouse)). DirectDIA search parameters included dynamic mass error for both precursor ions and fragment ions, trypsin enzyme specificity, carbamidomethylation as a fixed variation, methionine oxidation and N-terminal acetylation as variable modifications, and two missed cleavages. Label-free protein identification used the default target decoy approach with a Pulsar 1% FDR threshold for PSM, peptide, and protein group. Global signal imputation and automatic local normalization were applied. Individual protein abundance was determined by the area under the curve at the fragment ion (MS2) levels (Table S2).

### 2.4 Statistical Analysis

Both DDA and DIA protein-level matrices were preprocessed prior to differential expression analysis. To address ambiguous protein assignments, entries were split into separate rows corresponding to each individual protein accession. To enable cross-platform comparison and downstream biological analysis (such as compartment analysis and pathway enrichment analysis), gene names were used as the primary “protein” identifier. For the Orbitrap Exploris 480 DDA data set, gene names were retrieved using the UniProt API, mapping UniProt IDs to their corresponding gene names. All data sets were log2-transformed with a pseudocount of one. Outlier detection was performed via principal component analysis and hierarchical clustering. This led to the removal of sample T1030_HO_F from the Orbitrap Exploris 480 DIA data set, which consistently clustered apart as exhibited (Figure S2). Differential protein expression between normoxia and hyperoxia (*n* = 6 per group, balanced for sex: 3 males and 3 females per group) was performed using limma R package (v3.58.1)^32^ under R version 4.3.1. A linear model was fitted to the data using a design matrix that included disease status and sex as covariates. Empirical Bayes moderation was applied to improve variance estimation, and a contrast was defined to compare control mice against hyperoxia mice (Table S3). Proteins were considered differentially expressed if they met the Benjamini–Hochberg adjusted *p*-value threshold < 0.05 and |Log_2_ FC| > 1.

### 2.5 Bioinformatic Analysis

To assess the subcellular distribution of identified and differentially expressed proteins in each data set, compartment enrichment analysis was performed using SubcellularRVis.^33^ The list of proteins of interest was uploaded to the platform and analyzed against a reference background of all detected proteins in the data set (Table S4, Figure S4). Pathway enrichment analysis was conducted using the EnrichR.^34^ Differentially expressed proteins were submitted, and the Reactome 2024 database was selected for pathway annotation. A significance threshold of adjusted *p*-value < 0.01 was applied to identify enriched pathways (Table S5). To assess concordance between platforms, a Pearson correlation analysis was performed using a linear model of log-10 transformed intensities of matched IDs from each pair of instruments. Power analysis estimation for each data set was performed using PowerExplorer R package (v1.6.0). ClueGO v2.5.10 ^35^/Cluepedia v1.5.10 ^36^ plugin from Cytoscape v3.10.3 ^37^ was used to define GO enrichment specificity to subclasses of DIA DEPs. For each DIA data set, we defined three subclasses of differentially expressed proteins based on their relationship with the 480 DDA data set: DDA significant (also differentially regulated in DDA), DDA detected (also detected in DDA, but not significantly dysregulated), or DDA undetected (not detected in DDA). We tested each data set subclass against the gene ontology (GO) molecular function reference set to identify GO term associations, using the following settings: adjusted *p* ≤ 0.05, kappa score ≥ 0.1, GO tree levels ranging from 1 to 8, and the GO term fusion feature to eliminate parent–child term redundancy. GO reports were exported (Table S6). Beyond traditional enrichment metrics, these reports also include cluster assignment columns (which breakdown the distribution of proteins for any given GO term, based on the cluster, i.e., subclass of origin).^35^ These include cluster (term specificity to the input subclass: NA, detected, and significant), genes (gene names in each term from each subclass), percentage (of genes from each subclass), and percentage adjusted to input size (of genes from each subclass).

## 3 Results and Discussion

The study aimed to compare the global proteomic profiling performance of four mass spectrometers. We aimed to characterize the key differences in (1) acquisition modes, namely, data-dependent acquisition (DDA) and data-independent acquisition (DIA), and (2) different MS instrument generations (the gold-standard Orbitrap Exploris 480 vs new-generation Orbitrap Astral & timsTOF Ultra) (Figure 1A).

**Figure 1:**
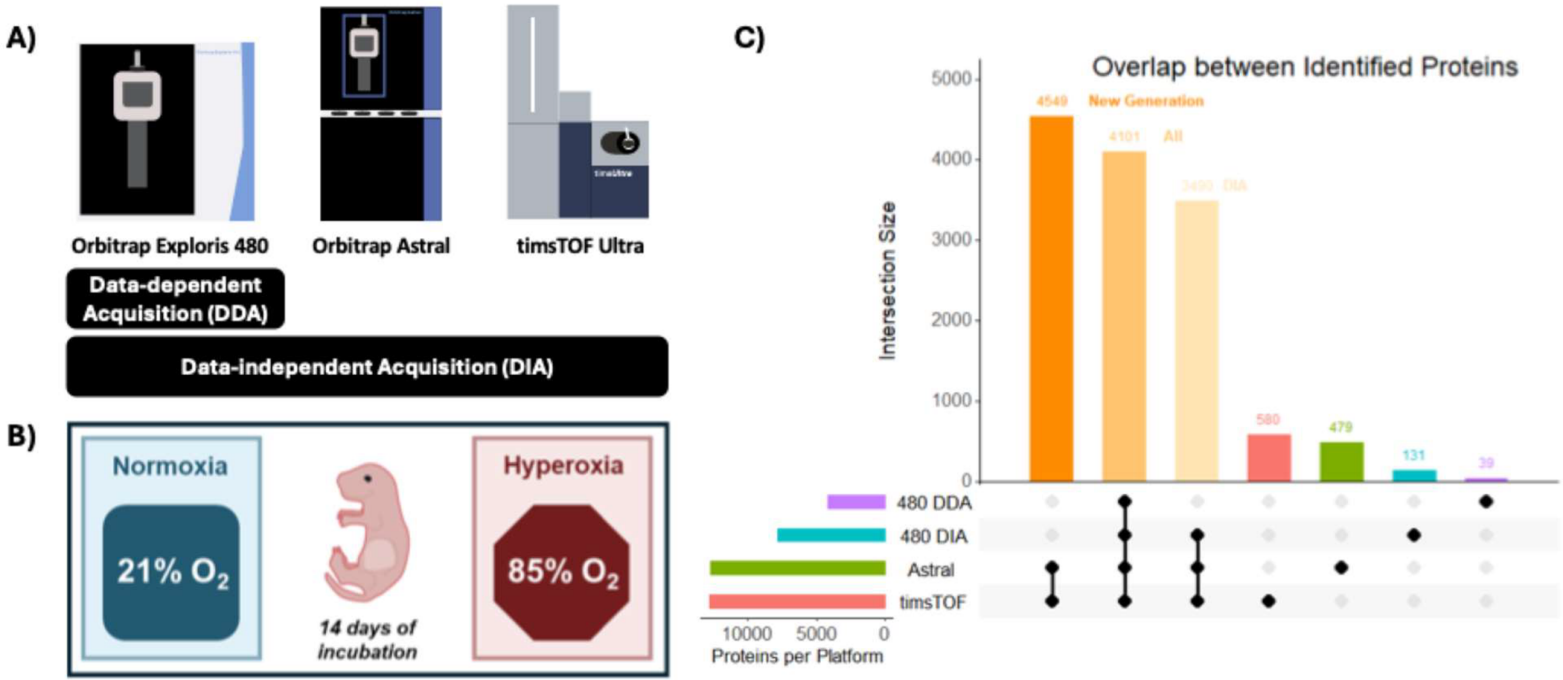
Study scheme and BPD model. (A) Schematic overview of MS instrumentation and data acquisition mode. (B) Mouse pups were incubated for 14 days in either normoxia or hyperoxia conditions. (C) Upset plot highlights the overlap among proteomes from different platforms (Orbitrap Exploris 480 DDA in purple, Orbitrap Exploris 480 DIA in blue, Orbitrap Astral in green, and timsTOF Ultra in coral). Mouse pup illustration (B) was sourced from SciDraw and adapted from Heath Robinson (licensed under a CC-BY 4.0 license).

We performed this comparison to provide practical insight into the relative strengths and trade-offs of each platform. To evaluate the platform performance on biologically relevant samples (e.g., in detecting phenotype-associated changes in protein expression), we performed quantitative label-free profiling using neonatal lung tissues from an animal model of bronchopulmonary dysplasia (BPD) (Figure 1B). Samples were collected from two experimental groups (normoxia (NO) and hyperoxia (HO)), each consisting of six mice, balanced for sex (three males and three females per group), and each sample was aliquoted for analysis across the instruments (Figure 1B). All samples were processed in parallel and analyzed under optimized but comparable instrument settings. Using a BPD model to compare MS performances across acquisition modes, we found that 4101 proteins were identified by all platforms. This meant that >99% of the proteome detected by Orbitrap Exploris 480 in DDA mode was recapitulated by all DIA modes (Figure 1A–C, Table S2). New-generation instruments identified 12,140 proteins out of all of the proteins identified in this study, reflecting a >90% concordance between them. As for DIA modes, they consistently identify 7591 proteins out of all of the proteins identified in this study, accounting for ∼57% of the data set (Figure 1C, Table S2).

### 3.1 Overview of Platform Settings and Global Performance Metrics

To provide an overview of each MS platform, we compiled the major platform features and performance metrics in Table 1.

**Table 1:** Overview of MS Platform Features

platform	Orbitrap Exploris 480 DDA	Orbitrap Exploris 480 DIA	Orbitrap Astral	timsTOF Ultra
sample prep	EasyPep MS Prep Kit (Thermo): 100 ng/uL aliquots in 0.1%FA			
digest load	600 ng	600 ng	175 ng	175 ng
LC system	Vanquish NanoUPLC	Vanquish NanoUPLC	Vanquish NanoUPLC	nanoElute2 LC
gradient time	180 min	60 min	60 min	52 min
software	PeaksStudio 10.6	Spectronaut 19	Spectronaut 19	Spectronaut 19
acquisition mode	DDA	Velocity DIA	DIA	dia-PASEF
imputation	random forest	global imputation	global imputation	global imputation
normalization	TIC normalization	local normalization^a^	local normalization^a^	local normalization^a^
precursors	33,322	104,903	302,593	340,004
peptides	30,092	85,930	238,606	253,558
% median CV peptide	50.99%	36.60%	29.40%	30.40%
protein	4200	7810	13492	13481
% median CV protein	33.10%	25.72%	18.75%	16.87%
sign proteins (#/%)^b^	304 (7.24%)	798 (10.21%)	1241 (9.20%)	920 (6.82%)
missingness (#/%)^c^	(592/3.66%)	(1642/4.57%)	(1322/2.37%)	(1148/2.19%)

a Local normalization: Spectronaut Local Normalization based on Callister et al. (2006).

b Significant proteins: adjusted *p*-value threshold < 0.05 and |Log_2_ FC| > 1.

c Percent missingness: Fraction of measurements not quantified.

Equal amounts of the digest (175 ng) were analyzed on the timsTOF Ultra and Orbitrap Astral using an optimized gradient for each instrument. These data were benchmarked against our standard 480 Orbitrap Exploris 480 DDA and DIA protocols, which analyze 600 ng and employ a 180 min gradient for DDA and a 60 min gradient for DIA. Table 1 highlights the analytical parameters and metrics we used in our benchmark analysis. To assess proteome coverage, we evaluated the number of precursors, nonredundant peptides, and proteins using a 1% FDR cutoff for protein and peptide identifications across all platforms. As anticipated, DIA acquisition on all instruments outperformed DDA coverage. This is attributed to the ability to comprehensively sequence all detectable peptides in any given run in DIA. Overall, we observed ∼200% improvement in precursor detection in Orbitrap Exploris 480 DIA, an ∼800% increase in Orbitrap Astral and ∼900% increase in timsTOF Ultra. The improved sensitivity in precursor detection translated to similar fold improvements in peptide identification (∼200% increase in DIA-Orbitrap Exploris 480, an ∼700% increase in Orbitrap Astral, and an ∼750% increase in timsTOF Ultra) and protein identification (∼90% increase in DIA-Orbitrap Exploris 480, an ∼220% increase in Orbitrap Astral, and an ∼220% increase in timsTOF Ultra). Due the rapid scan times and smaller acquisition windows on both Orbitrap Astral and timsTOF Ultra, DIA on these instruments quantified approximately ∼725% more than Orbitrap Exploris 480 in DIA acquisition mode. Moreover, comparable improvements in the completeness of data were observed in both Orbitrap Astral and timsTOF Ultra versus Orbitrap Exploris 480.

### 3.2 How Does Increased Protein Detectability Impact Biological Coverage

While increasing the proteome coverage is important, we wanted to understand how these numbers really affect the ability of proteomics to probe a biological model. We started by comparing the ability of each data set to probe each subcellular compartment. The analysis showed a consistent increase of coverage across all subcellular components, with Orbitrap Exploris 480 DDA showing an average compartment coverage of ∼30%, Orbitrap Exploris 480 DIA ∼45%, and new generation ∼66% (Figure 2A,B, Table S4, Figure S4).

**Figure 2:**
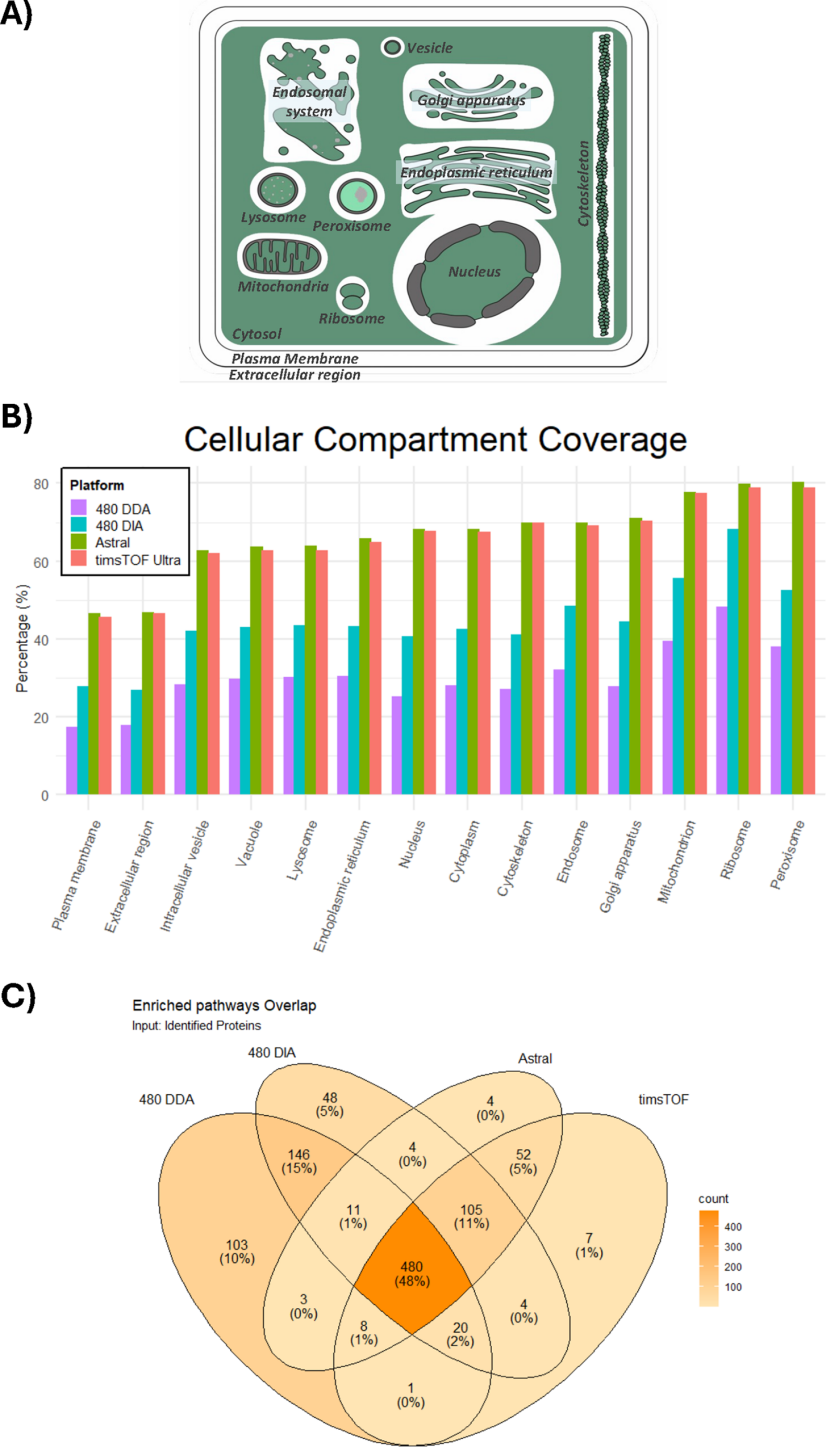
How does increased protein detectability impact biological coverage. (A) Graphical representation of cellular compartment coverage. (B) Bar plot represents the cellular compartment coverage (%) from each proteome (Orbitrap Exploris 480 DDA in purple, Orbitrap Exploris 480 DIA in blue, Orbitrap Astral in green, and timsTOF Ultra in coral). (C) Overlap of the reactome pathway enriched terms (adjusted *p*-value threshold < 0.01).

These results show that DIA-enabled proteome depth is not being driven by the ability to detect proteins localized to one or more specific subcellular compartments. To complement our understanding of how well these data sets probe a biological model, we explored whether bigger data sets translated into a more complete pathway annotation. Overall, DIA methods show a significant increase on both total (from ∼41 to ∼59% average coverage) and significantly enriched pathways (from ∼58 to 75% average coverage, FDR < 1%) (Tables 2 and S5) compared to Orbitrap Exploris 480 DDA. Next-generation platforms further boost the average pathway coverage of the total pathways to ∼75% and from enriched pathways to ∼90% (Tables 2 and S5). Furthermore, the Venn diagram highlights how the enriched pathways (FDR < 1%)) overlap between each data sets.

**Table 2:** Pathway Enrichment Analysis of Proteomes across Platforms

	**total pathways**		**enriched pathways (adj P-val <0.01)**	
**MS platforms**	**# pathways**	**avg % coverage**	**# pathways**	**avg % coverage**
Orbitrap Exploris 480 DDA	1939	40.70%	771	58.09%
Orbitrap Exploris 480 DIA	2035	58.73%	818	75.25%
Orbitrap Astral	2077	75.14%	666	90.04%
timsTOF Ultra	2073	75.70%	676	89.94%

We found DIA methods to capture ∼90% of the pathways enriched in 480 Orbitrap Exploris in DDA acquisition mode. Furthermore, 48% of all of the enriched pathways overlap among all data sets (Figure 2C). The enriched pathways associated with the new-generation instrumentation show an overlap of >90%, once again highlighting a consistent probe of the proteome. We hypothesize that the moderate percentages of pathways enriched exclusively in the 480 Orbitrap Exploris in DDA and DIA data sets are due to different levels of proteome coverage (∼4 k for Exploris DDA, ∼8 k for Exploris DIA, and ∼13 k for new-generation instruments) yielding different hierarchies of the same pathway term.

### 3.3 New-Generation Instruments Capture More Biological Variance

Next, we analyzed whether the measurements from each platform differently captured the structure of the data. Principal component analysis (PCA) revealed clear separation between control and hyperoxia groups in PC1, across all four mass spectrometry platforms (Figure 3A–D). While Orbitrap Exploris 480 in DDA mode achieved biological group separation with moderate explained variance (PC1:27.66%), switching to DIA on the same instrument markedly increased the variance explained by biological groups (PC1:41.86%) (Figures 3A–D and S5). Both Orbitrap Astral and timsTOF Ultra platforms further improved in discriminatory power, with PC1 explaining ∼46% of the variance in both cases. Other PCs capture a consistent variance structure, as evidenced by the comparable distribution of explained variance for >PC2 (Figure S5).

**Figure 3:**
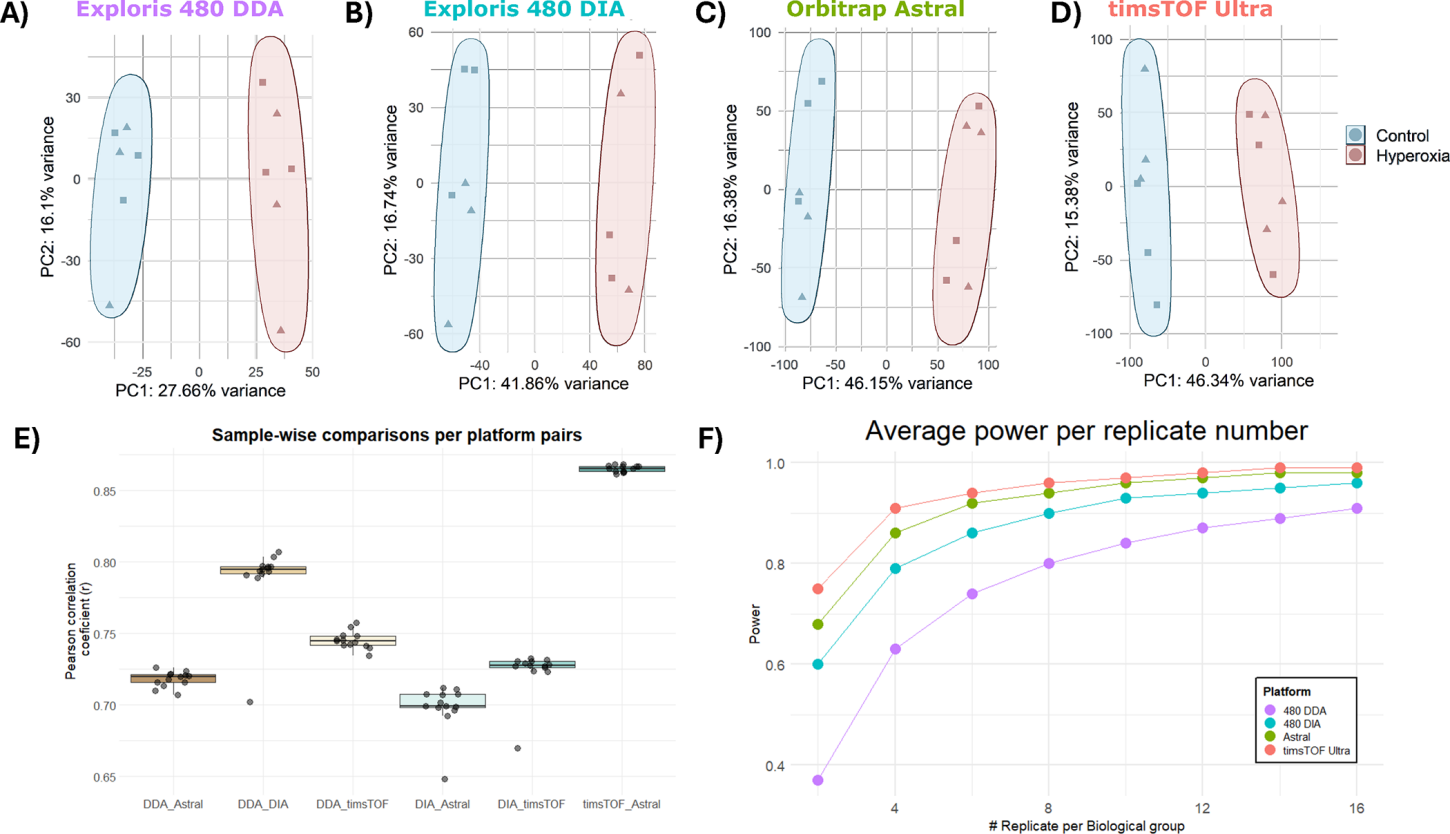
New-generation platforms capture more biological variance. (A–D) Principal component analysis of the lung proteome across all platforms; the biological groups (control and hyperoxia) are separated through principal component 1. (E) Average Pearson correlation coefficient comparison between pairs of platforms. (F) Line graph highlights the average power for increasing biological group sizes estimated for all platforms (Orbitrap Exploris 480 DDA in purple, Orbitrap Exploris 480 DIA in blue, Orbitrap Astral in green, and timsTOF Ultra in coral).

Next, we performed a sample-wise correlation and averaged the Pearson correlation coefficient values for all pairs of instruments. This analysis evaluates how each sample proteome profiling correlates between instruments (Figures 3E, and S6–S11). The best average sample-wisecorrelation among any platform pair was between the new-generation platforms (timsTOF Ultra vs Orbitrap Astral: ∼0.87), followed by the Orbitrap Exploris 480 with different acquisition modes (Orbitrap Exploris 480 DDA vs DIA: ∼0.79) (Figures S6–S11). timsTOF exhibited slightly higher correlation with both DDA and DIA acquisition modes in Orbitrap Exploris 480 (DDA vs timsTOF: ∼0.75, DIA vs timsTOF ∼0.73) as compared to Orbitrap Astral (DDA vs Astral: ∼0.72, DIA vs Astral: ∼0.70) (Figure S6–11). These results are consistent with Huang et al. 2025;^21^ in this study, authors show pairwise correlation of LFQ intensities between the Orbitrap Astral and timsTOF Ultra 2 for different HeLa amounts, yielding a Pearson correlation coefficient ranging from 0.74 to 0.86.

### 3.4 Statistical Power

In parallel, we performed sample size estimations to understand whether the higher ability of new-generation instruments to capture biological signal gathers enough statistical power to effectively reduce sample size needs (Figures 1 and 3F). Power calculations were performed using the PowerExplorer R package with the following settings: log_2_ FC = 1, *a* = 0.1, 90% power with ROTS permutation testing (B = 1000, ST = 150). Our analysis showed that to reach a gold standard threshold of 90% power,^38^ 15, eight, five, and four replicates are needed per biological groups, respectively, for Orbitrap Exploris 480 DDA, Orbitrap Exploris 480 DIA, Orbitrap Astral, and timsTOF Ultra, respectively (Figure 3F). Overall, new-generation platforms cut recommended sample size by ∼66%, which improves ethical balance, experimental duration, and cost.

### 3.5 Capturing Differentially Expressed Proteins across Platforms

To assess how proteome coverage impacts the identification of differentially expressed proteins, we used limma with empirical Bayes estimation. We defined differentially expressed proteins (DEPs) as those meeting the combined threshold of a Benjamini–Hochberg adjusted *p*-value < 0.05 and |Log_2_ FC| > 1. We found that higher coverage of the proteome translated into more DEPs, with 480 Orbitrap Exploris increasing from 304 to 798 DEPs when switching to DIA mode and with next-generation instruments yielding 920 (timsTOF Ultra) and 1241 DEPs (Orbitrap Astral) (Tables 3 and S3).

**Table 3:** Summary of Differentially Expressed Proteins (DEPs) across Different Statistical Thresholds

		**adj. *p*-val < 0.05**	**adj. *p*-val < 0.01**	**adj. *p*-val < 0.05** **& B > 0**	**adj. *p*-val < 0.05** **& |log FC| > 1**
Orbitrap Exploris 480 DDA	sign up	481	255	181	144
	sign down	456	253	178	160
	non sign	3263	3692	3841	3894
Orbitrap Exploris 480 DIA	sign up	676	365	252	455
	sign down	1068	598	408	343
	non sign	6315	7096	7399	7261
Orbitrap Astral	sign up	3864	2897	1832	756
	sign down	3610	2760	1734	485
	non sign	6157	7974	10,065	12,390
timsTOF Ultra	sign up	4348	3370	2114	609
	sign down	3476	2625	1636	311
	non sign	5865	7694	9939	12,769
% of total data set	480 Exploris DDA	22.31	12.1	8.55	7.24
	480 Exploris DIA	21.64	11.95	8.19	9.90
	Orbitrap Astral	54.83	41.5	26.16	9.10
	timsTOF Ultra	57.16	43.79	27.39	6.72

We further explored whether the identified differential expressed proteins capture known canonical molecular features of BPD. We looked at the expression of surfactant protein D, which is central to both innate immune defense and surfactant homeostasis, whose dysregulation contributes to impaired lung maturation in BPD.^39–42^ All approaches identified surfactant protein D to be significantly upregulated in the hyperoxia group (480 DDA: log_FC_ = 2.79, 480 DIA: log_FC_ = 2.65, Astral: log_FC_ = 2.70, and timsTOF: log_FC_ = 2.54), which is concordant with previous literature^42^ (Table S3).

To understand how different DEP data set sizes impact biological annotation, we performed subcellular component and pathway enrichment analysis by restricting our attention to differentially expressed proteins. Subcellular compartment analysis showed that the mitochondrion and extracellular regions were enriched in differentially expressed proteins (FDR < 1%) for all platforms (Figure 4A,B, Table S4, Figure S4); this is expected as mitochondrial^43,44^ and extracellular matrix dysfunction^45,46^ is central to BPD pathogenesis.

**Figure 4:**
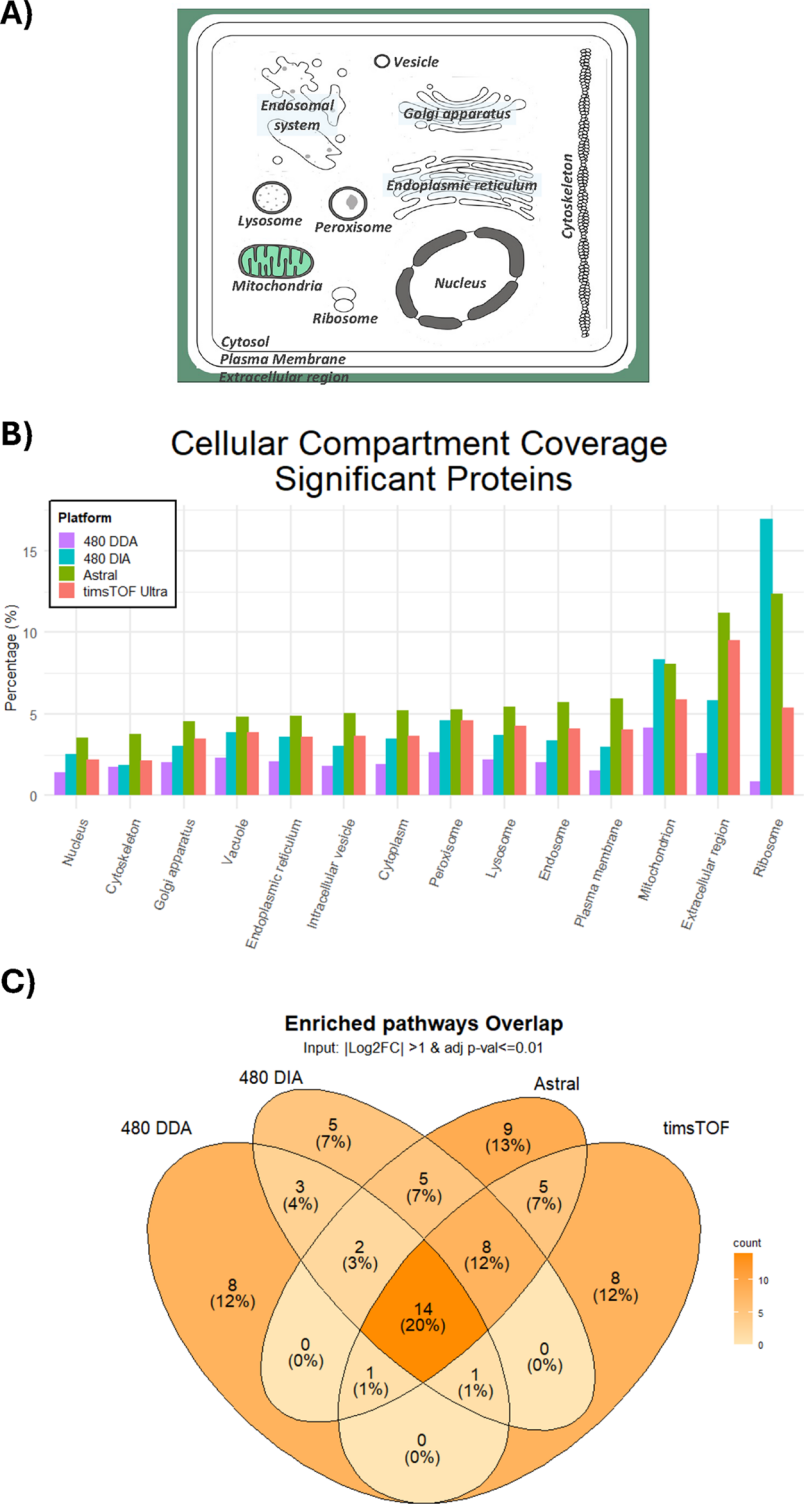
Capturing differentially expressed proteins. (A) Graphical representation of cellular compartment coverage of DEP data sets. (B) Bar plot highlights the cellular compartment coverage (%) from each DEP data set (Orbitrap Exploris 480 DDA in purple, Orbitrap Exploris 480 DIA in blue, Orbitrap Astral in green, and timsTOF Ultra in coral). (C) Overlap of the reactome pathway enriched terms (adjusted *p*-value threshold < 0.01).

Overall, subcellular compartment enrichment analysis showed a similar pattern across subcellular compartments with higher DEP data sets yielding higher coverages, except for 480 Orbitrap Exploris DIA favoring the mitochondrion and ribosome (Figure 4B, Table S4, Figure S4). Looking at enriched reactome pathways, DIA methods showed a significant increase in enriched pathway coverage (from ∼13 to 25% average coverage, FDR < 1%) compared to Orbitrap Exploris 480 DDA (Tables 4 and S5). Furthermore, the Venn diagram highlights how the enriched pathways (FDR < 1%) overlap between each data sets, with 20% of all of the enriched pathways overlapping among all data sets (Figure 4C). Enriched pathways associated with new-generation instrumentation showed an overlap of ∼55%.

**Table 4:** Pathway Enrichment Analysis of Differentially Expressed Proteins across Platforms

	**total pathways**		**enriched pathways (adj *p*-val < 0.01)**	
**MS platforms**	**# pathways**	**avg % coverage**	**# pathways**	**avg % coverage**
Orbitrap Exploris 480 DDA	930	5.83%	28	12.80%
Orbitrap Exploris 480 DIA	1103	7.93%	37	29.21%
Orbitrap Astral	1334	10.12%	44	25.61%
timsTOF Ultra	1172	8.36%	36	23.14%

Despite a >90% overlap in pathways enriched in the proteomes of new-generation instruments (Figure 3C, Table S5), this concordance is reduced to ∼55% when looking at pathways enriched in DEP subsets (Figure 4C, Table S5). Altogether, these data suggest that annotations generated from DEP show a higher degree of variance.

### 3.6 DIA Proteome Depth Provides Completeness of Biology Rather Than Uniqueness

Next, we explored whether the expanded protein coverage enabled by DIA acquisition modes provides additional biological insights compared to DDA. To prioritize biological relevance, we exclusively explored how DIA differentially expressed proteins mapped to the 480 DDA data set. We started by annotating the DIA data sets based on their relationship with the 480 DDA data set: DDA significant (also differentially regulated in DDA), DDA detected (also detected in DDA, but not significantly regulated), or DDA undetected (not detected in DDA). Out of the 798 proteins differentially expressed in the 480 DIA data set, 122 were also significant in DDA, 186 were detected in DDA, and 468 were undetected in DDA (Figure 5A).

**Figure 5:**
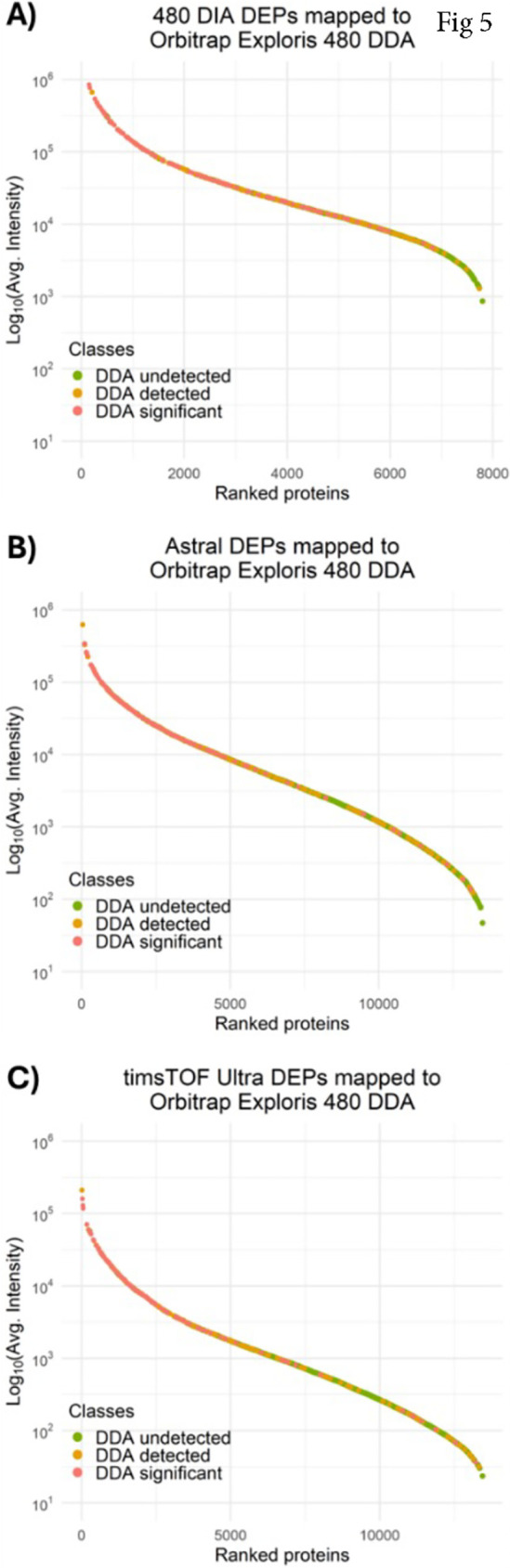
DIA proteome depth provides completeness of biology rather than uniqueness. A proteome dynamic range of differentially expressed proteins from Orbitrap Exploris 480 DIA (A), Orbitrap Astral (B), and timsTOF Ultra (C) annotated to the Orbitrap Exploris 480 DDA (coral: also differentially expressed in DDA, orange: also detected in DDA, green: undetected in DDA).

Out of the 1241 proteins differentially expressed in the Orbitrap Astral data set, 118 were also significant in DDA, 173 were detected in DDA, and 915 were undetected in DDA (Figure 5B). Lastly, out of the 920 proteins differentially expressed in the timsTOF data set, 102 were also significant in DDA, 136 were detected in DDA, and 682 were undetected in DDA (Figure 5C). Overall, ∼70% of differentially regulated proteins in DIA data sets are not detected by DDA, ∼18% are detected in DDA, and only ∼12% are differentially expressed proteins in both data sets. Beyond numbers, we wanted to assess whether the biology being captured by each subset is redundant or unique. We used ClueGO^35–37^ to perform GO analysis comparing the enriched terms from each subset. ClueGO uses GO term fusion, eliminating parent–child term redundancy, enabling reliable comparison across the three subclasses, and allowing the assignment of subclass specificity. For all DIA data sets, the enriched GO terms showed no subclass specificity (Table S6), meaning the GO molecular functions enriched in DDA significant, DDA detected, and DDA undetected classes are redundant and therefore no unique biology is being gained by DIA. This data alongside the previously shown increase in pathway coverage in DIA data sets for all identified proteins (Figure 2, Table 2) suggests that DIA provides more completeness of information. We conclude that DDA approaches can capture the biology of a given model, and DIA approaches greatly improve the completeness of this information.

## 4 Conclusions

Data-independent acquisition coupled with the improved sensitivity and scan rates of next-generation instruments results in a much higher ability to identify peptides and proteins. Our data shows that these extended proteomes boost biological annotation completeness. Furthermore, these more complete proteomes help better resolve biological group structure and increase statistical power, with estimates suggesting next-generation platforms reduce biological replicate needs by ∼66%. Next-generation instrumentation also shows remarkable concordance in probing the proteome; however, variance emerges when capturing differential expression. Inherent differences in technical details pose a challenge when comparing performance. One caveat of our study was the use of modestly different columns, gradients, and sample amounts, which prevents instrument comparison from LC performance. However, this was a conscious trade-off, as our primary goal was to explore the capabilities of many platforms available in the market. Further, this work represents a snapshot in time for the proteomics field, where we expect continued advances in key areas like ion optics,^47^ faster data acquisition speeds, and enhanced bioinformatics frameworks.^48–51^ The future looks promising, with anticipated applications including spatial proteomics and other techniques paralleling advances seen in genomics, positioning the field for increasingly comprehensive systems biology ascertainment.

## Supplementary Material















## Data Availability

The data sets supporting the conclusions of this article are available in publicly available repositories. The Orbitrap Exploris 480 mass spectrometry proteomics data relative to label-free mass spectra have been deposited to the ProteomeXchange^52^ Consortium via the PRIDE^53^ partner repository with the data set identifier PXD057442 and can be found at 10.6019/PXD057442. The DIA data sets Orbitrap Exploris 480 DIA, Orbitrap Astral, and timsTOF Ultra mass spectrometry data relative label-free mass spectra have been deposited to the ProteomeXchange^52^ Consortium via the MASSIVE partner repository with the data set identifier MSV000099422 and can be found at ftp://massive-ftp.ucsd.edu/v11/MSV000099422/

## Notes

timsTOF Ultra windows scheme (Figure S1); clustering analysis to identify outliers (Figure S2); evaluation of quantitative performance (Figure S3); subcellular compartment enrichment analysis graphical visualization (Figure S4); combined scree plots (Figure S5); and sample-wise correlation between instrument pairs (Figures S6–11) (PDF)
timsTOF Ultra dia-PASEF isolation list (XLSX)
Protein-level reports for all instruments (XLSX)
Limma reports for all data sets (XLSX)
Subcellular compartment enrichment analysis tables for all data sets (XLSX)
Reactome analysis tables for all data sets (XLSX)
GO specificity analysis (XLSX)

## Abbreviations

Astral: asymmetric track lossless analyzer
BPD: bronchopulmonary dysplasia
DDA: data-dependent acquisition
DIA: data-independent acquisition
FDR: false discovery rate
GO: gene ontology
HCD: higher energy collision dissociation
HO: hyperoxia
HR: high resolution
MS: mass spectrometry
NO: normoxia
tims: trapped ion mobility spectrometry
TIC: total ion chromatogram
TOF: time-of-flight
FC: fold-change
LC: liquid chromatography
MS/MS: tandem mass spectrometry
PASEF: parallel accumulation-serial fragmentation
PSM: peptide-spectrum match
ROS: reactive oxygen species
UPLC: ultraperformance liquid chromatography.
