# Supplementary material for: What Does Next-Generation Mass Spectrometry Offer for Proteomics? A Comprehensive Platform Comparison
Source: J Proteome Res. 2026 Mar 26;25(4):1929–40. doi: 10.1021/acs.jproteome.5c01007 (PMC13054874; doi:10.1021/acs.jproteome.5c01007)
Supplement: Supplementary file 1 [file pr5c01007_si_001.pdf]

What does next generation Mass Spectrometry offer for proteomics? A comprehensive platform comparison

Filipa Blasco Tavares Pereira Lopes[1,2], Daniela Schlatzer[1], Tara Sudhadevi[3], Anantha Harijith[3], Marzieh Ayati[4], Mehmet Koyutürk[1,5], Mark R. Chance[1,2,]*

1 Center for Proteomics and Bioinformatics, Case Western Reserve University, Cleveland, OH

2 Department of Nutrition, Case Western Reserve University, Cleveland, OH

3 Department of Pediatrics, Case Western Reserve University, Cleveland, OH

4 Department of Computer Science, University of Texas Rio Grande Valley

5 Department of Computer and Data Sciences, Case Western Reserve University, Cleveland, OH

* Corresponding author

Figure S1 – Graph visualizing dia-PASEF windows isolation width scheme for timsTOF Ultra.

Figure S2 – Outlier Identification in Orbitrap Exploris 480 DIA. PCA identified an outlier (**A**), which was confirmed to be sample T030_HO_F by hierarchical clustering analysis (**B**); Graphs **C** and **D** show the effects of sample T030_HO_F sample removal in the Orbitrap Exploris 480 DIA dataset.

Figure S3 – Evaluation of quantitative performance of biological replicates. Histogram showing the distribution of missing values (**A**) and violin plot of median % CV for each platform (**B**).

Figure S4 – Graphical visualization of subcellular compartment enrichment analysis using Subcellular RVis from: Exploris 480 DDA proteome (**A**) and DEP (**B**), Exploris DIA proteome (**C**) and DEP (**D**), Orbitrap Astral proteome (**E**) and DEP (**F**), timsTOF Ultra proteome (**G**) and DEP (**H**) datasets.

Figure S5 – Combined Scree plot of all datasets colored according to its source dataset (Exploris 480 DIA in purple, Exploris 480 DIA in blue, timsTOF Ultra in salmon, and Orbitrap Astral in green).

Figure S6 - Sample-wise Pearson correlation plots between Orbitrap Exploris 480 in DDA and DIA modes. Scatter plots of Log10 Orbitrap Exploris DDA vs DIA protein intensities with fitted linear regression, r-squared, and Pearson’s r highlighted in red.

Figure S7 - Sample-wise Pearson correlation plots between Orbitrap Exploris 480 in DDA and Orbitrap Astral. Scatter plots of Log10 Orbitrap Exploris DDA vs Orbitrap Astral protein intensities with fitted linear regression, r-squared, and Pearson’s r highlighted in red.

Figure S8 - Sample-wise Pearson correlation plots between Orbitrap Exploris 480 in DDA and timsTOF Ultra. Scatter plots of Log10 Orbitrap Exploris DDA vs timsTOF Ultra protein intensities with fitted linear regression, r-squared, and Pearson’s r highlighted in red.

Figure S9 - Sample-wise Pearson correlation plots between Orbitrap Exploris 480 in DIA and Orbitrap Astral. Scatter plots of Log10 Orbitrap Exploris DIA vs Orbitrap Astral protein intensities with fitted linear regression, r-squared, and Pearson’s r highlighted in red.

Figure S10 - Sample-wise Pearson correlation plots between Orbitrap Exploris 480 in DIA and timsTOF Ultra. Scatter plots of Log10 Orbitrap Exploris DIA vs timsTOF Ultra protein intensities with fitted linear regression, r-squared, and Pearson’s r highlighted in red.

Figure S11 - Sample-wise Pearson correlation plots between Orbitrap Astral and timsTOF Ultra. Scatter plots of Log10 Orbitrap Astral vs timsTOF Ultra protein intensities with fitted linear regression, r-squared, and Pearson’s r highlighted in red.

Table S1 – timsTOF Ultra dia-PASEF isolation list (xlsx)

Table S2 – Protein-level reports for all instruments (xlsx)

Table S3 – Limma reports for all datasets (xlsx)

Table S4 – Subcellular compartment enrichment analysis tables for all datasets (xlsx)

Table S5 – Reactome analysis tables for all datasets (xlsx)

Table S6 – GO specificity analysis (xlsx)

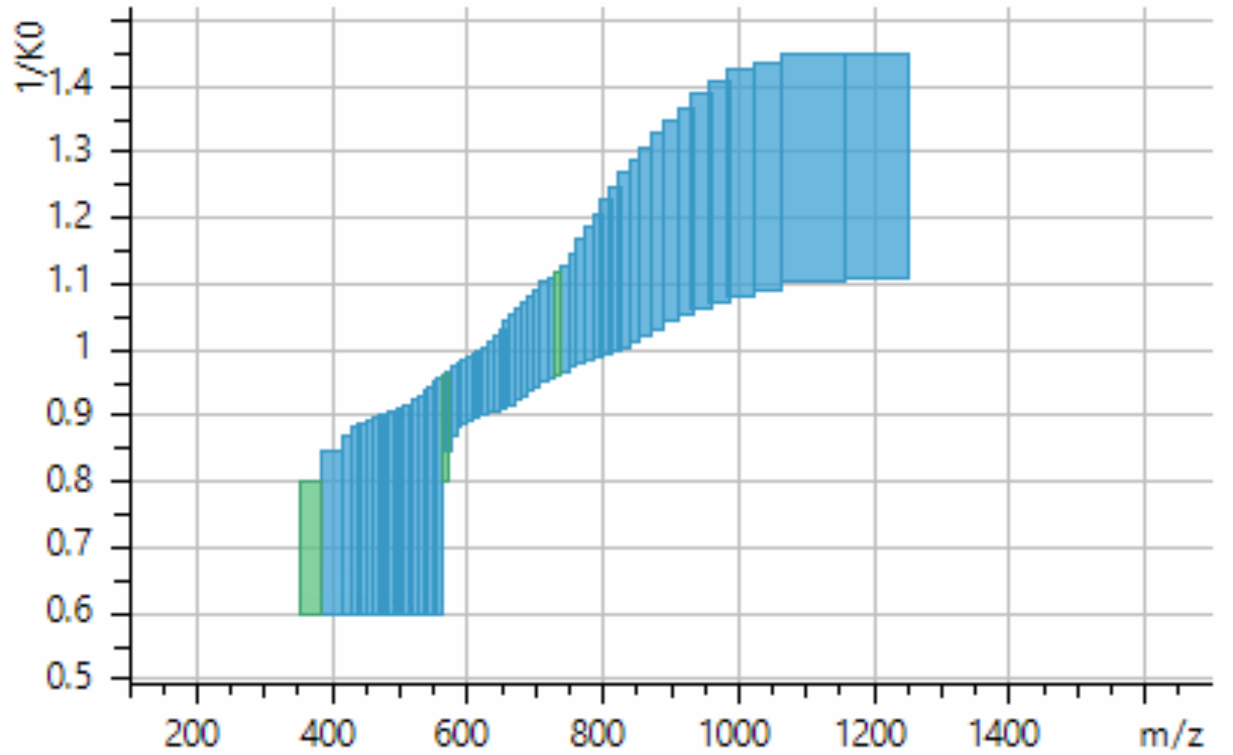

Figure S1 – Graph visualizing dia-PASEF windows isolation width scheme for timsTOF Ultra.

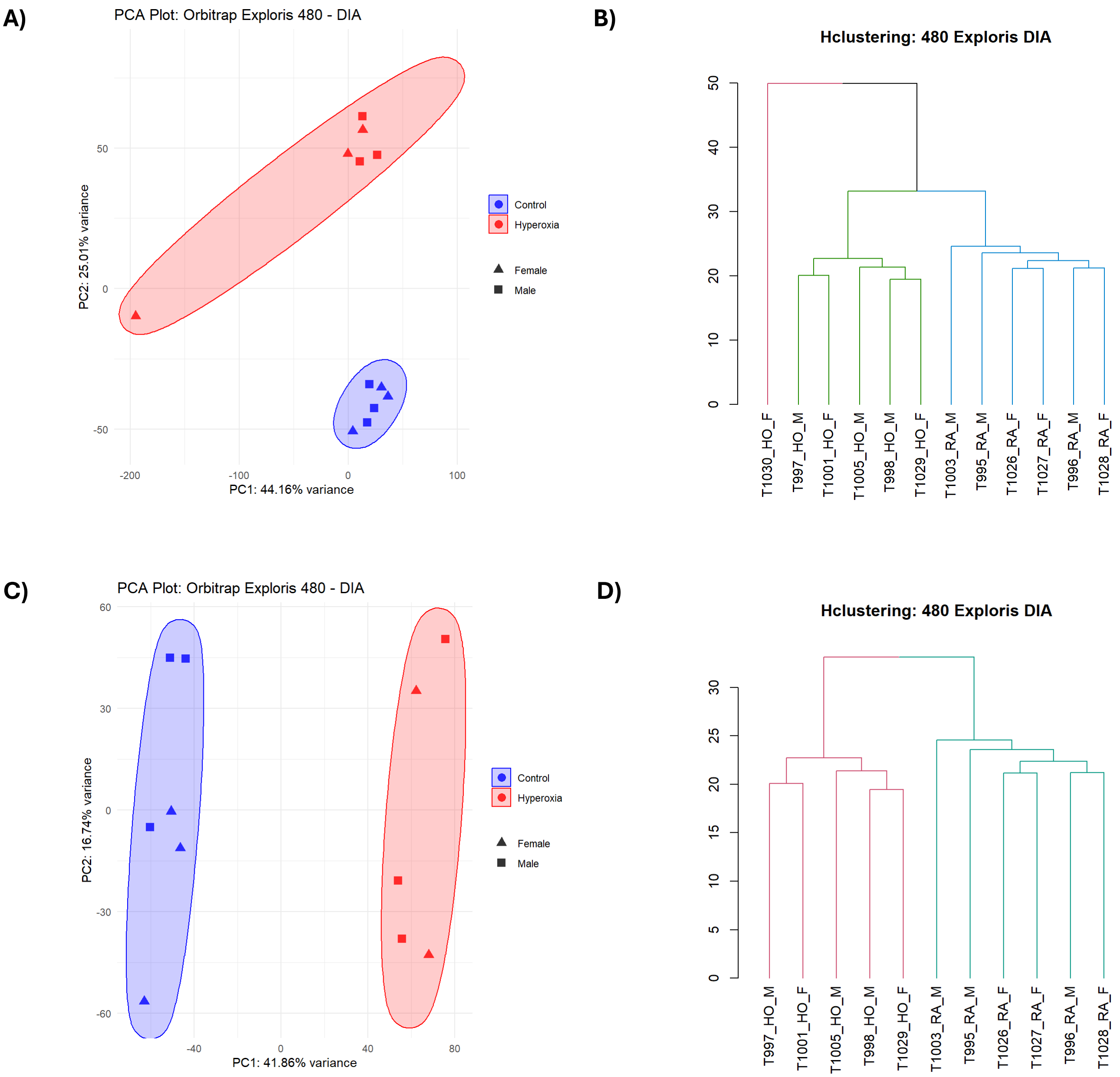

Figure S2 – Outlier Identification in Orbitrap Exploris 480 DIA. PCA identified an outlier (**A**), which was confirmed to be sample T030_HO_F by hierarchical clustering analysis (**B**); Graphs **C** and **D** show the effects of sample T030_HO_F sample removal in the Orbitrap Exploris 480 DIA dataset.

A)

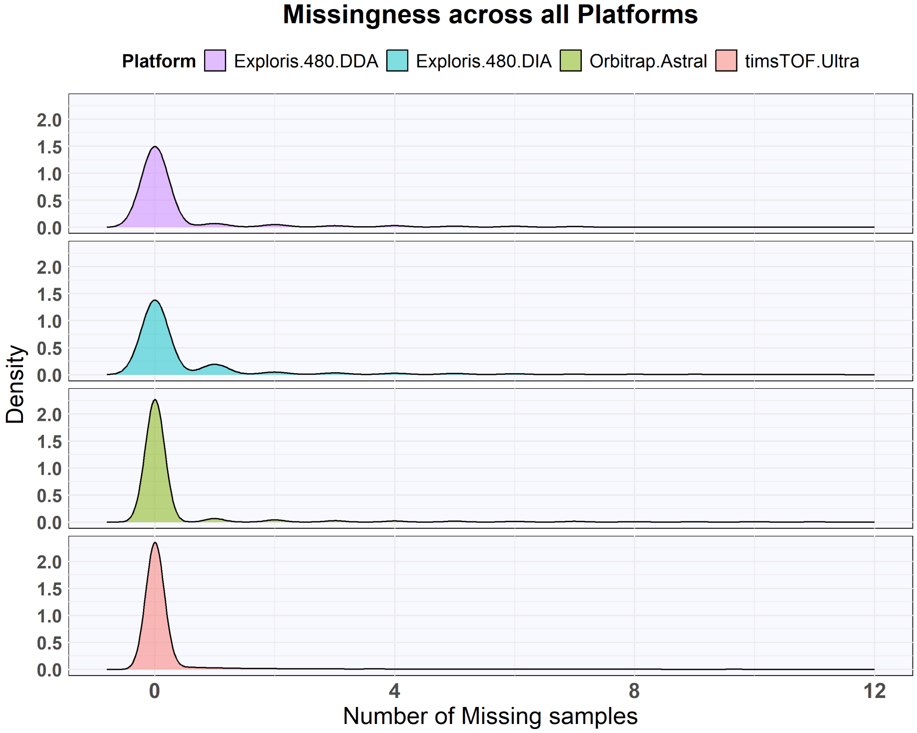

B)

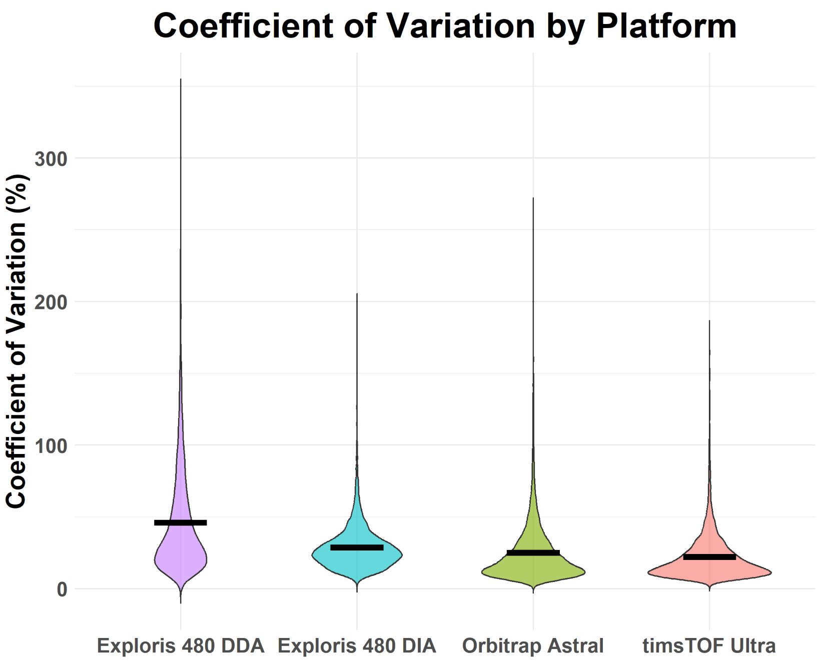

Figure S3 – Evaluation of quantitative performance of biological replicates across platforms. Histogram showing the distribution of the number of missing values (**A**) and violin plot of median % CV for each platform (**B**).

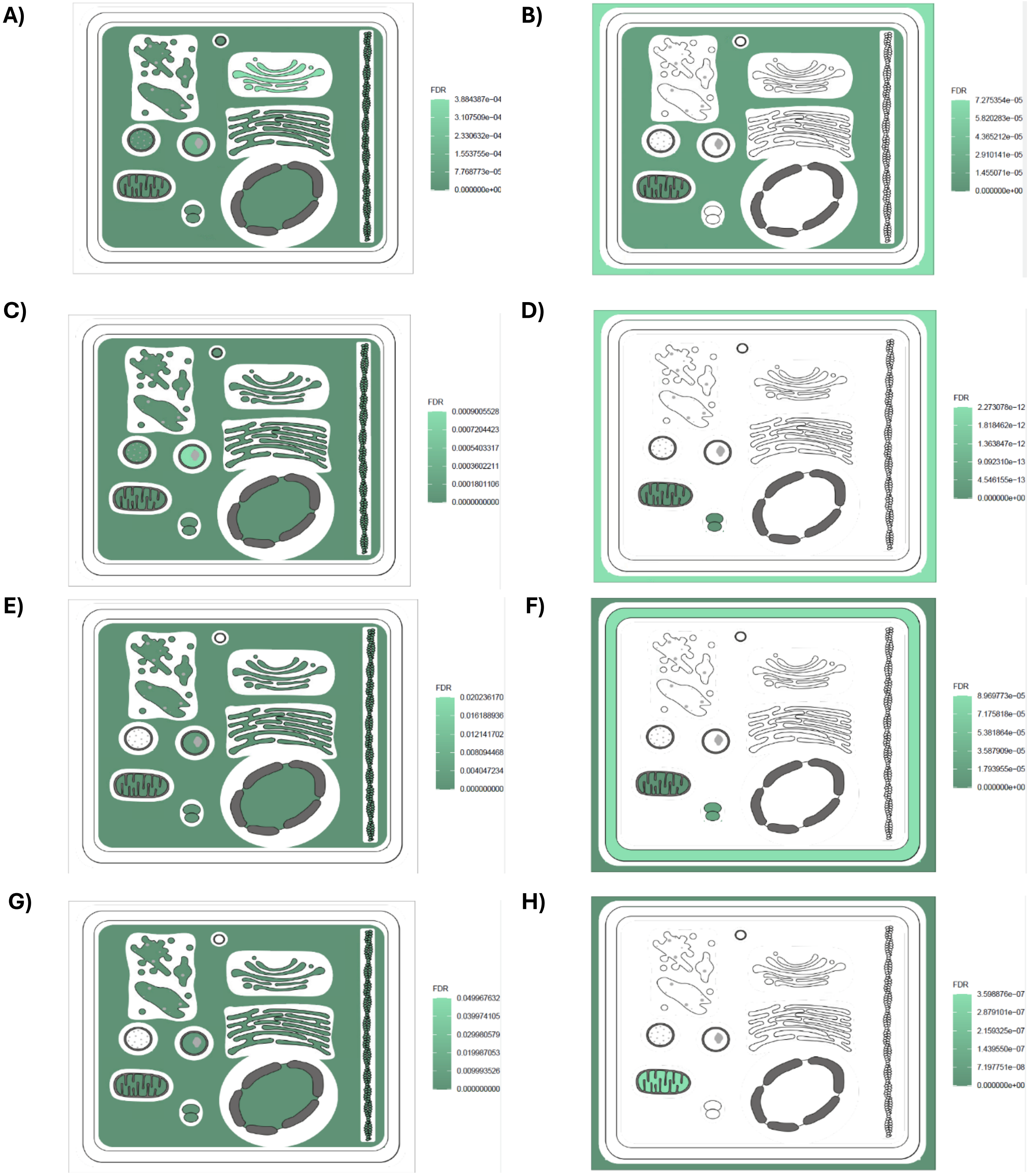

Figure S4 – Graphical visualization of subcellular compartment enrichment analysis using Subcellular RVis from: Orbitrap Exploris 480 DDA proteome (**A**) and DEP (**B**), Orbitrap Exploria DIA proteome (**C**) and DEP (**D**), Orbitrap Astral Proteome (**E**) and DEP (**F**), timsTOF Ultra proteome (**G**) and DEP (**H**) datasets.

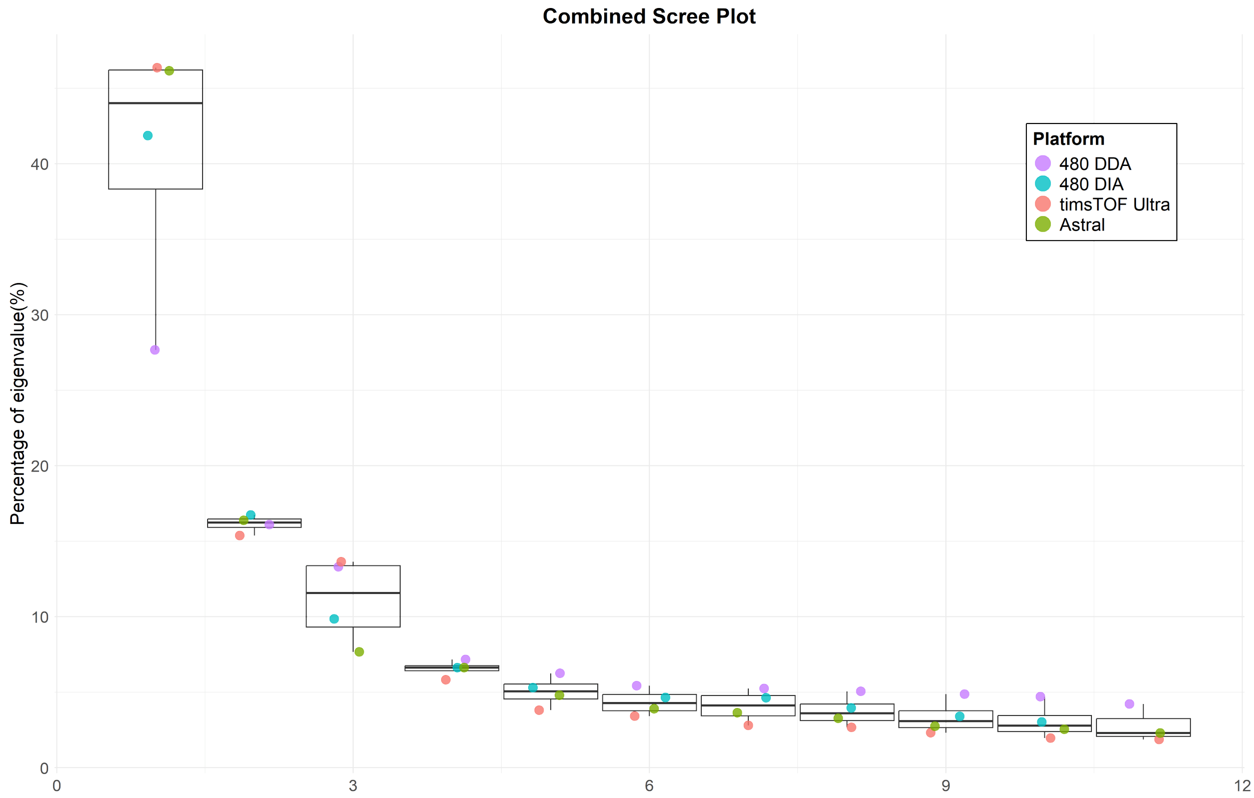

Figure S5 – Combined Scree plot of all datasets with dots colored according to its source dataset (Orbitrap Exploris 480 DIA in purple, Orbitrap Exploris 480 DIA in blue, timsTOF Ultra in salmon, and Orbitrap Astral in green).

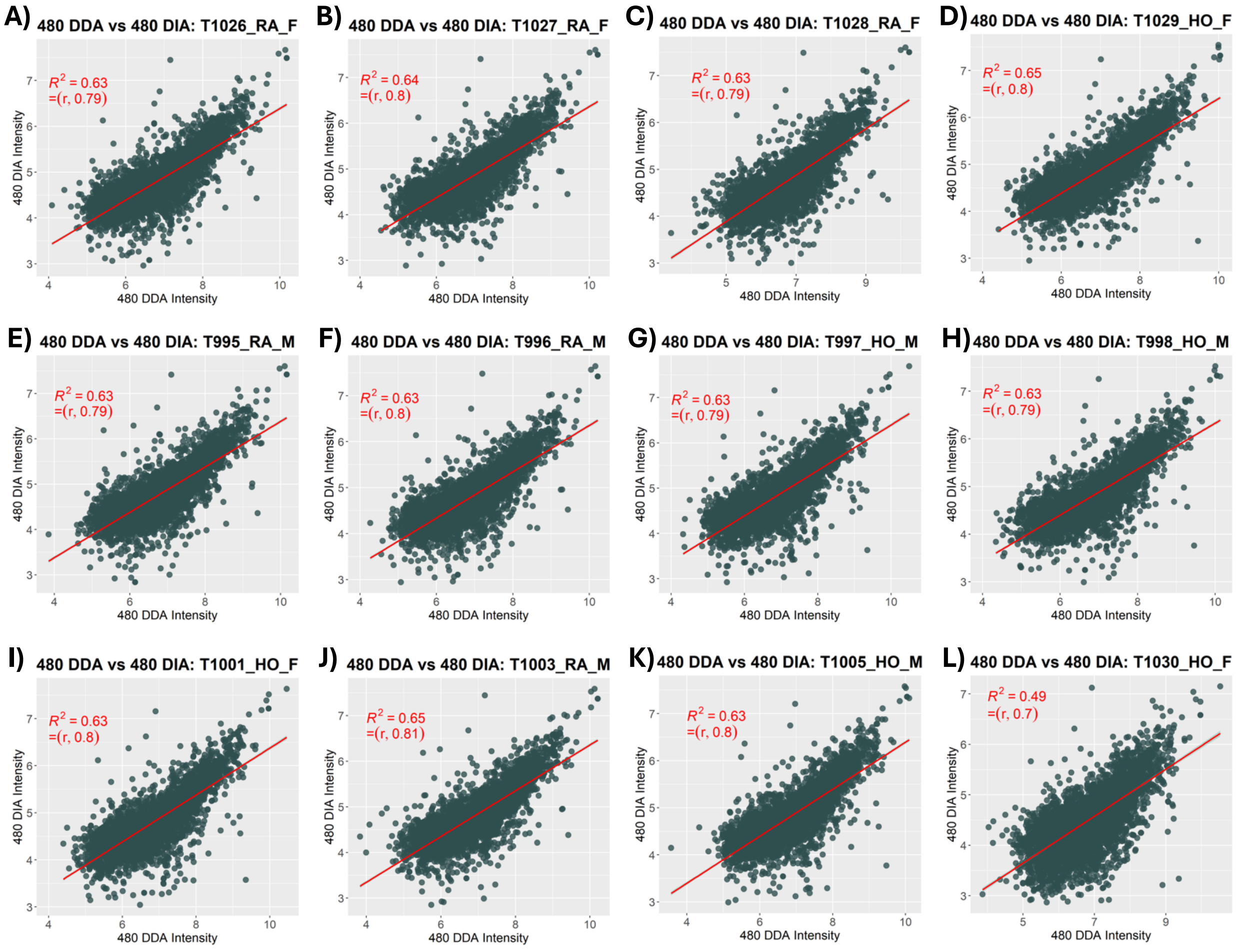

Figure S6 - Sample-wise Pearson correlation plots between Orbitrap Exploris 480 in DDA and DIA modes. Scatter plots of Log10 Orbitrap Exploris DDA vs DIA protein intensities with fitted linear regression, r-squared, and Pearson's r highlighted in red.

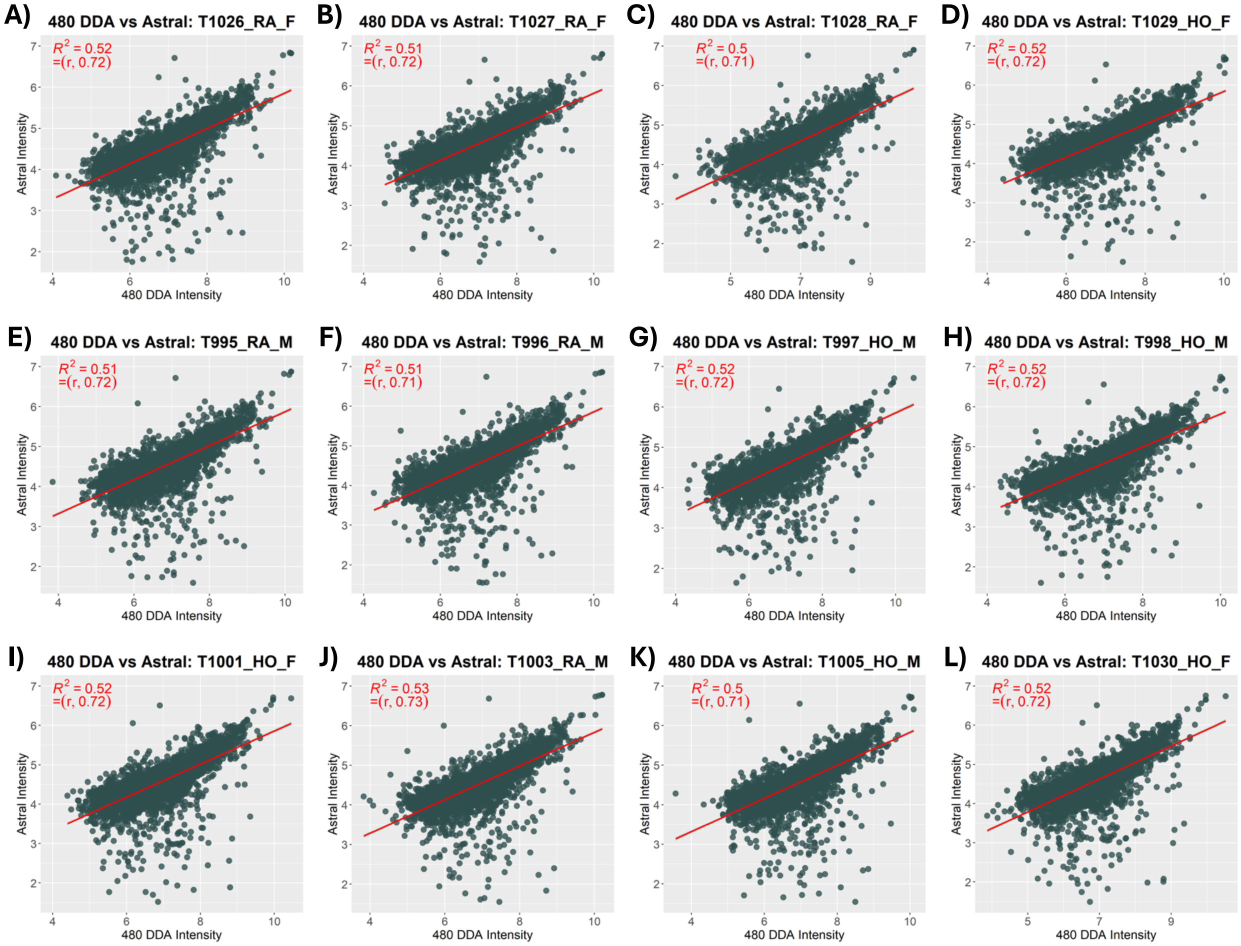

Figure S7 - Sample-wise Pearson correlation plots between Orbitrap Exploris 480 in DDA and Orbitrap Astral. Scatter plots of Log10 Orbitrap Exploris DDA vs Orbitrap Astral protein intensities with fitted linear regression, r-squared, and Pearson's r highlighted in red.

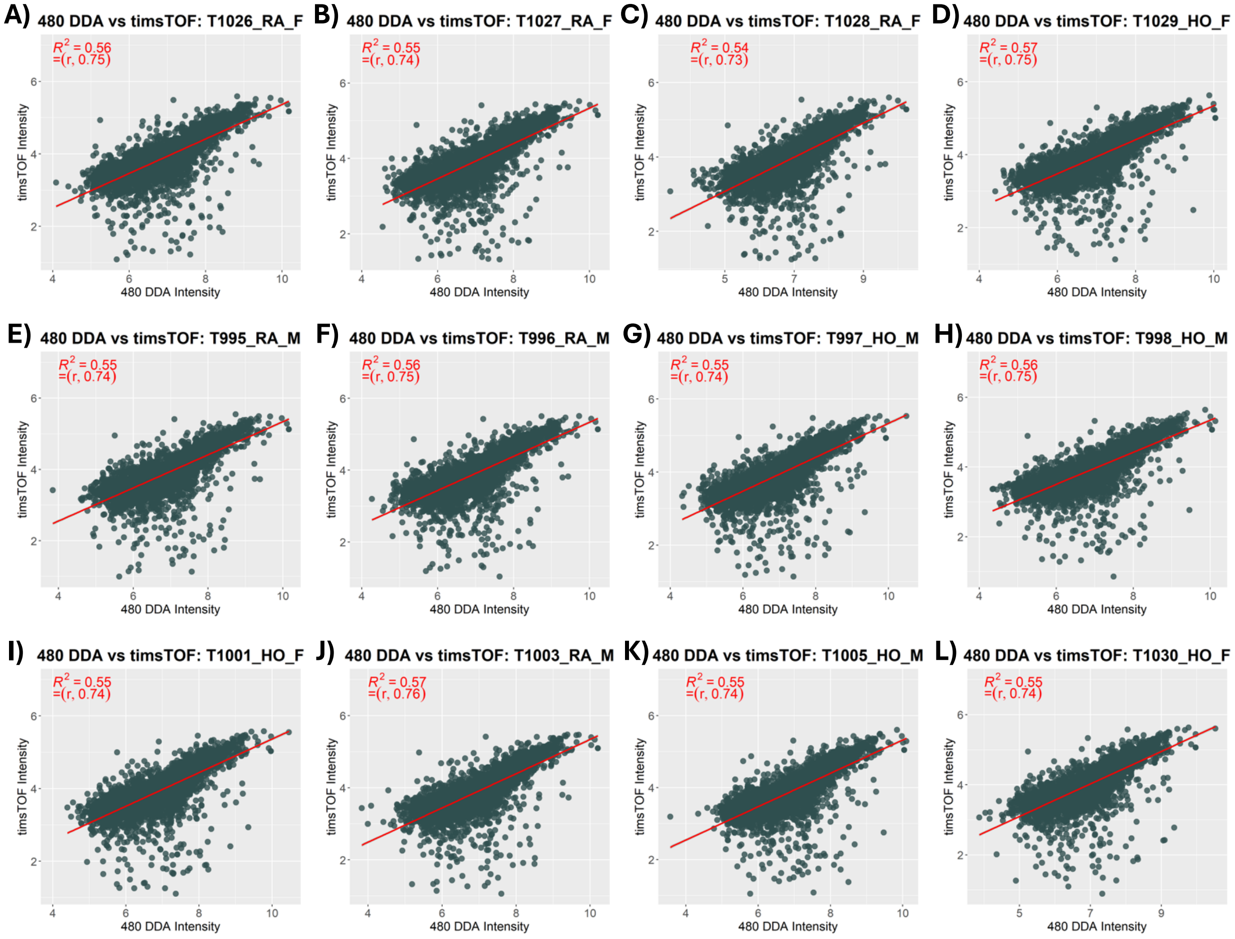

Figure S8 - Sample-wise Pearson correlation plots between Orbitrap Exploris 480 in DDA and timsTOF Ultra. Scatter plots of Log10 Orbitrap Exploris DDA vs timsTOF Ultra protein intensities with fitted linear regression, r-squared, and Pearson's r highlighted in red.

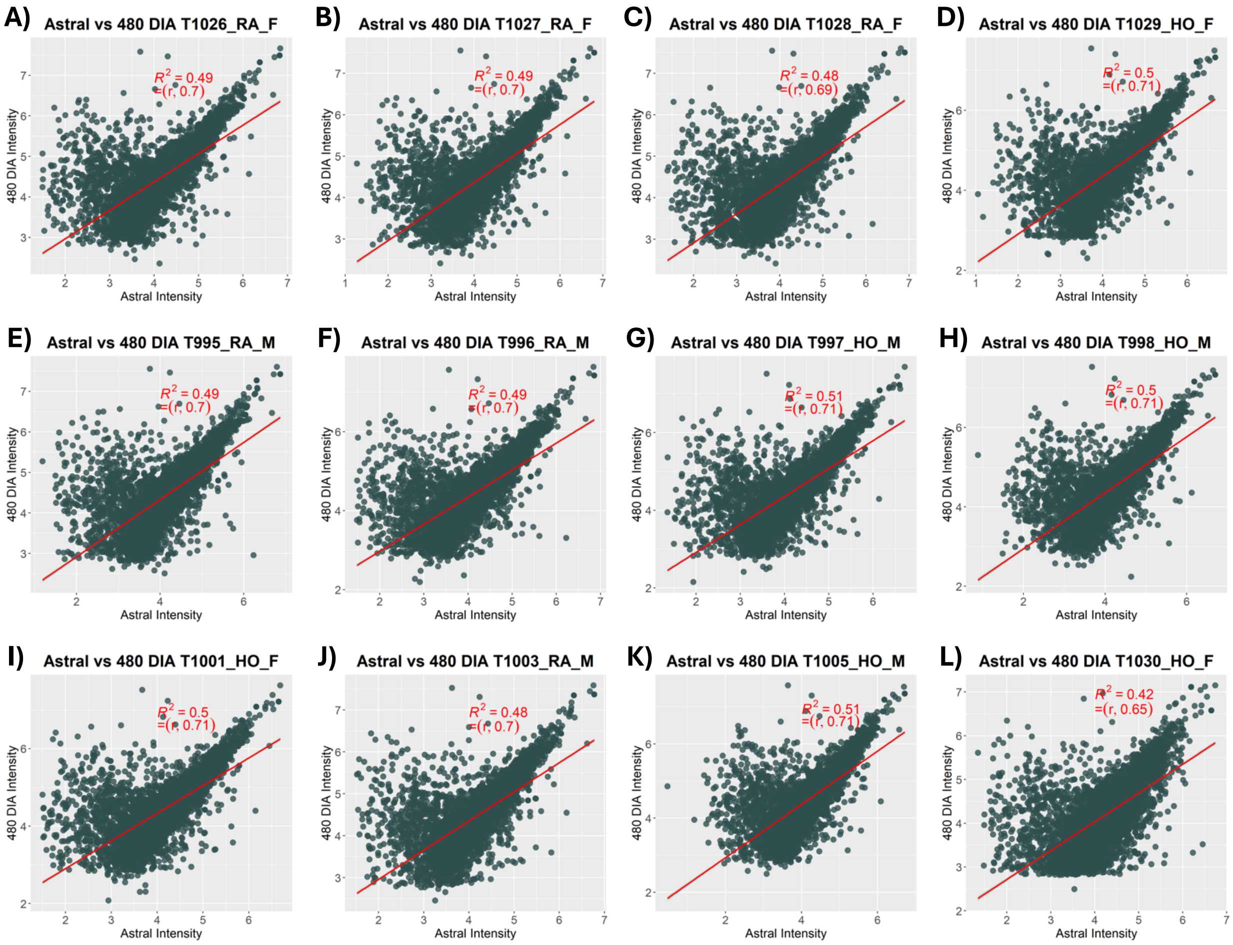

Figure S9 - Sample-wise Pearson correlation plots between Orbitrap Exploris 480 in DIA and Orbitrap Astral. Scatter plots of Log10 Orbitrap Exploris DIA vs Orbitrap Astral protein intensities with fitted linear regression, r-squared, and Pearson's r highlighted in red.

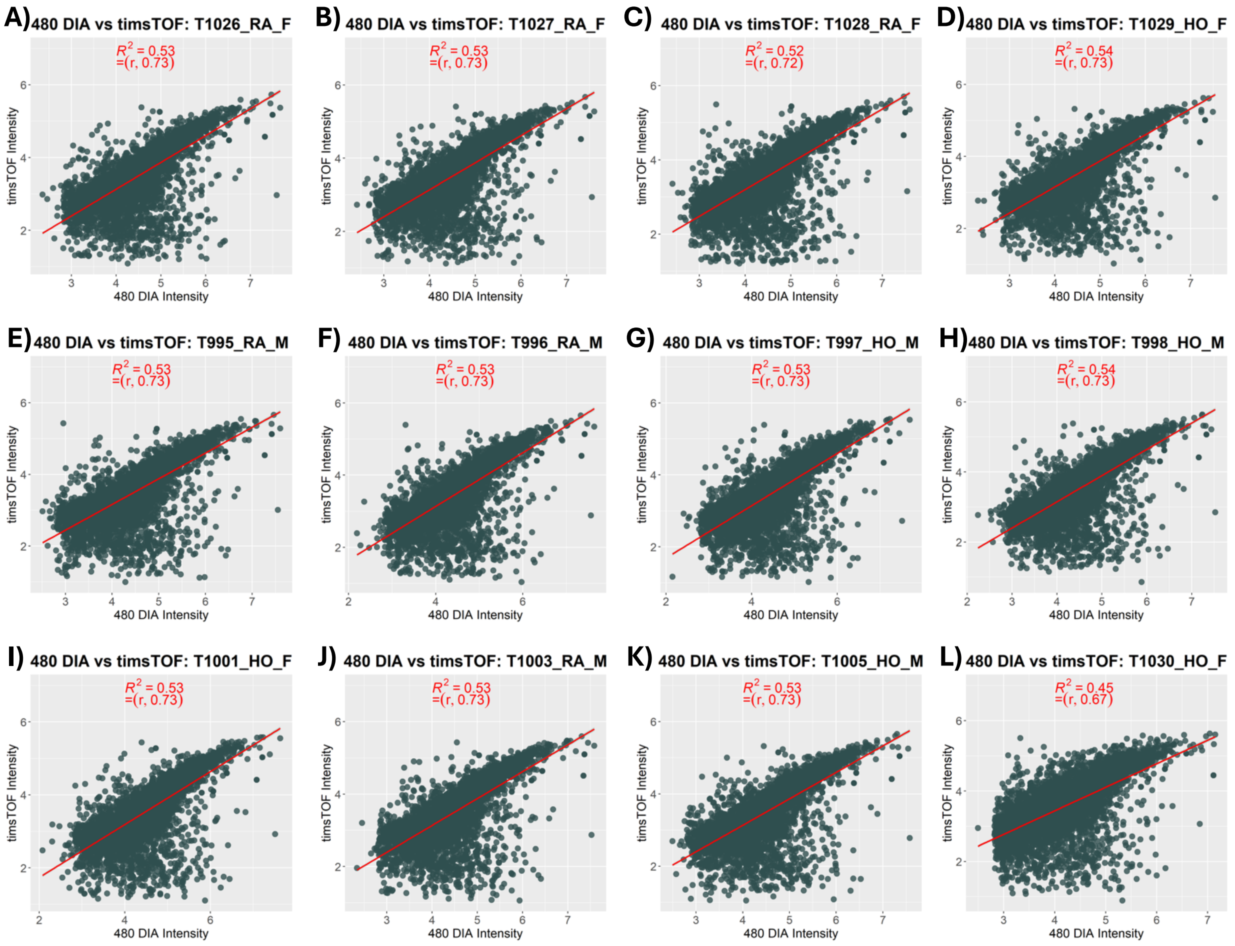

Figure S10 - Sample-wise Pearson correlation plots between Orbitrap Exploris 480 in DIA and timsTOF Ultra. Scatter plots of Log10 Orbitrap Exploris DIA vs timsTOF Ultra protein intensities with fitted linear regression, r-squared, and Pearson's r highlighted in red.

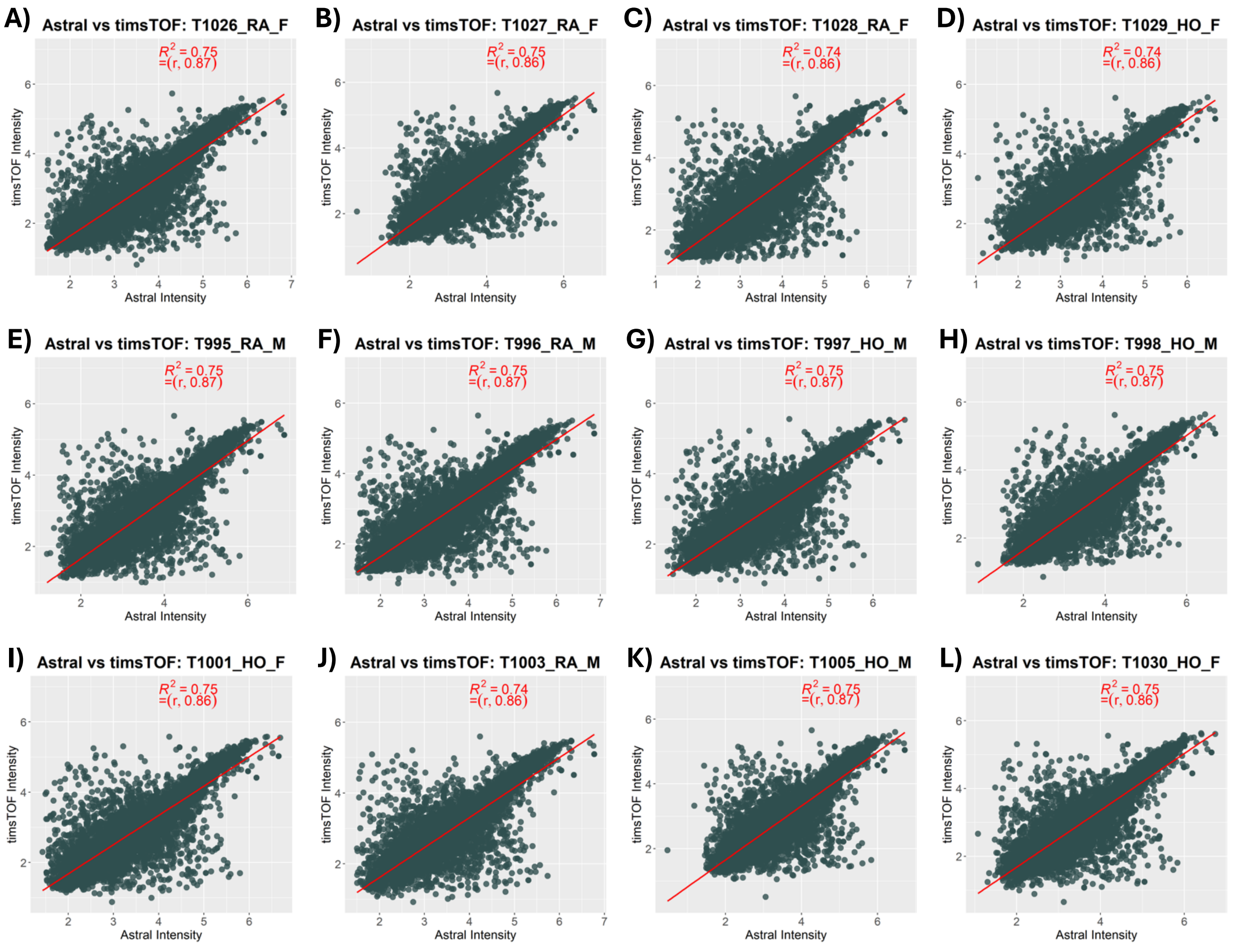

Figure S11 - Sample-wise Pearson correlation plots between Orbitrap Astral and timsTOF Ultra. Scatter plots of Log10 Orbitrap Astral vs timsTOF Ultra protein intensities with fitted linear regression, r-squared, and Pearson's r highlighted in red.
